# A Simple Target Interception Task as Test for Activities of Daily Life Performance in Older Adults

**DOI:** 10.3389/fnins.2019.00524

**Published:** 2019-05-27

**Authors:** Alix L. de Dieuleveult, Sander I. B. Perry, Petra C. Siemonsma, Anne-Marie Brouwer, Jan B. F. van Erp

**Affiliations:** ^1^Predictive Health Technologies, Netherlands Organisation for Applied Scientific Research (TNO), Leiden, Netherlands; ^2^Perceptual and Cognitive Systems, Netherlands Organisation for Applied Scientific Research (TNO), Soesterberg, Netherlands; ^3^Human Media Interaction, University of Twente, Enschede, Netherlands; ^4^Fysiotherapie Dekker, Amstelveen, Netherlands; ^5^Clinical Epidemiology and Biostatistics, University of Amsterdam, Amsterdam, Netherlands; ^6^University of Applied Sciences for Physiotherapy (THIM), University for Physiotherapy, Nieuwegein, Netherlands; ^7^Department of Physiotherapy, University of Applied Sciences Leiden, Leiden, Netherlands

**Keywords:** aging, elderly, sensory integration, interception task, activities of daily living

## Abstract

Previous research showed that a simple target interception task reveals differences between younger adults (YA) and older adults (OA) on a large screen under laboratory conditions. Participants intercept downward moving objects while a horizontally moving background creates an illusion of the object moving in the opposite direction of the background. OA are more influenced by this illusory motion than YA. OA seem to be less able to ignore irrelevant sensory information than YA. Since sensory integration relates to the ability to perform Activities of Daily Living (ADL), this interception task can potentially signal ADL issues. Here we investigated whether the results of the target interception task could be replicated using a more portable setup, i.e., a tablet instead of a large touch screen. For YA from the same, homogeneous population, the main effects were replicated although the task was more difficult in the tablet set-up. After establishing the tablet’s validity, we analyzed the response patterns of OA that were less fit than the OA in previous research. We identified three different illusion patterns: a (large) illusion effect (indicating over integration), a reverse illusion effect, and no illusion effect. These different patterns are much more nuanced than previously reported for fit OA who only show over integration. We propose that the patterns are caused by differences in the samples of OA (OA in the current sample were older and had lower ADL scores), possibly modulated by increased task difficulty in the tablet setup. We discuss the effects of illusory background motion as a function of ADL scores using a transitional model. The first pattern commences when sensory integration capability starts to decrease, leading to a pattern of over-integration (illusion effect). The second pattern commences when compensatory mechanisms are not sufficient to counteract the effect of the background motion, leading to direction errors in the same direction as the background motion (reverse illusion). The third pattern commences when the task requirements are too high, leading OA to implement a probabilistic strategy by tapping toward the center of the screen.

## Introduction

The development of new health technologies and advancements in medicine have helped extending life expectancy of the global population (Dey, 2017). This increased life expectancy has led to a rise of the population of 60 years old and older, considered as older adults (OA) by the World Health Organization (Dey, 2017). In order to help solve future societal challenges and decrease for instance health care costs, age-related changes in physical abilities and functioning need to be studied.

Assisting OA to live independently for a longer time can help to reduce the age-related costs for the society and to increase their quality of life. In order to live independently, an individual needs to be able to perform both the basic (Katz et al., 1963) and instrumental (Lawton and Brody, 1969) activities of daily living (ADL), such as dressing and shopping. To do so, OA need a good level of mobility (Lowry et al., 2012) and need to be able to properly integrate the sensory information from the surroundings (Lowry et al., 2012). Sensory integration is known to change during the lifespan (Mozolic et al., 2012; Freiherr et al., 2013; de Dieuleveult et al., 2017). In a recent review (de Dieuleveult et al., 2017), we concluded that OA seem to integrate as much information as possible in their surroundings without properly weighing the information, thus also using irrelevant or unreliable information (Berard et al., 2012; Brodoehl et al., 2015; de Dieuleveult et al., 2017). Also, the addition of a dual task was shown to decrease the performance of OA to a greater level than that of YA indicating that OA seem to be less able to compensate for the increased attentional and sensory demand (Redfern et al., 2001; Mahboobin et al., 2007; Redfern et al., 2009; Bisson et al., 2014; de Dieuleveult et al., 2017).

Vision is essential for goal-directed reach movements toward moving targets (Brouwer et al., 2002, 2003; Kavcic et al., 2011; Brenner and Smeets, 2015). There are two ways to collect information to judge a target’s direction of motion. Both require the integration of multisensory information. The first way is by using the actual motion of the target in space (visual information), and the information about changes in the eye’s orientation in the head and changes in the orientation of the head (proprioceptive information) (Nakayama, 1985; Schweigart et al., 2003). The second way is by assuming that the surrounding remains static and use the relative motion of the target’s retinal image and its surrounding to estimate the target’s motion in space (visual and vestibular information). The background is then interpreted as optic flow due to the participant’s own motion (Brenner and van den Berg, 1994). If the background is moving, relying on relative motion will lead to systematic errors. In this case, refraining from relying on relative motion would be beneficial to increase precision. In an earlier study, Berard et al. (2012) found that old age affects the ability to down-regulate the influence of such visual information in a walking task. In an original experiment (de Dieuleveult et al., 2018), we investigate whether this effect generalizes to another paradigm, namely the paradigm of Brouwer et al. (2003) that used the Duncker illusion (or induced motion) (Duncker, 1929). The object appears to move differently due to movement in its surrounding (Soechting et al., 2001; Zivotofsky, 2004). We showed that OA were more affected by the illusion of motion created by a horizontally moving background than YA when trying to intercept disappearing targets on a large screen. OA’s interception points (called taps) were more deviated to the left for a right background motion and to the right for a left background motion than those of YA. These results could reflect a reduced ability to ignore or downregulate irrelevant sensory information and/or a greater reliance on vision because of unreliable somatosensory and proprioceptive systems (de Dieuleveult et al., 2018). If the ability to ignore or downregulate irrelevant sensory information is indeed reduced in OA, our interception task may be a useful tool to diagnose sensory integration problems that could underlie (future) problems in ADL.

Aging-related decline in perception and multisensory integration has been studied before, but the relation between this decline and ADL performance and its possible interaction with dual tasks is less well researched. If a reliable relation can be shown, the measure of multisensory integration may be used as an early diagnostic and/or predictive tool for ADL problems in individual OA. Such a tool is clinically relevant since decline in ADL can be slowed down or prevented using different exercise approaches targeting specific ADL problems. These approaches are already available and commonly used in clinical practice, for example strength training (Hazell et al., 2007), functional training (Liu et al., 2014), and balance training (Bellomo et al., 2009). Training of multisensory integration in the OA population has been developed as well (de Vreede et al., 2005; Setti et al., 2014; Merriman et al., 2015). However, no diagnostic tool assessing multisensory integration deficits related to the ADL exists (de Dieuleveult et al., 2017). Consequently, existing training approaches are blind to potential sources of the deficit, and training cannot be specifically tailored to individual OA’s multisensory integration issues during daily life. Self-report ADL questionnaires such as the NEADL can suffer from bias in memory and social demands and are not suited to find the cause of the ADL problem. The interception task may be able to do so in a direct and fun way, assessing a person’s sensory integration functioning by means of performance rather than self-report. It could be a good starting point to develop a clinically useful diagnostic toolkit of multisensory integration issues related to ADL. This could help clinicians to better tailor training programs to the individual’s underlying problems. An important step toward the development of a clinical tool is to develop and validate a mobile set-up of the task, that is practical and affordable and can be used for OA with different degrees of ADL performance.

The current interception task was adjusted from its original large screen (49 inch) set-up to a smaller tablet (12,3 inch) set-up in order to increase clinical applicability: a smaller setup is more convenient for clinical practice and could also be transported to measure individuals that are less fit and cannot easily travel to a clinician. The experiment reported here was roughly the same as in de Dieuleveult et al. (2018). YA and OA intercepted disappearing targets moving downwards on the tablet with a horizontally moving background. The interception task was performed either with or without one of two secondary tasks. The secondary tasks were chosen to impinge on different processes that are expected to relate to sensory perception and integration. The first one mainly disturbed proprioception, which is highly involved in postural stability. Postural stability is necessary for motor control, coordination and steadiness during the performance of ADL (Ghai et al., 2017). The second kind of dual task was mainly cognitive. Cognitive deficits have been shown to interfere with motor performance, tasks that were automatic (such as walking) require more cognitive attention with increasing age (Lima et al., 2015). OA are more prone to falls when they are trying to walk and perform a second task at the same time A larger background effect when adding dual tasks might reveal compensatory mechanisms that normally help to decrease deficits caused by the earlier reported over integration in OA. Additionally, the use of dual tasking in training programs has been shown to improve postural stability, notably for OA (Ghai et al., 2017).

First, this experiment aims at investigating whether we can replicate the results found in our original experiment (de Dieuleveult et al., 2018) with a small, portable tablet instead of a large screen. Therefore, we test a sample of YA from the same population as in the original experiment, and we hypothesize that the effect of the moving background would be similar for YA in both experiments (H1). The second aim was to investigate task performance of OA with problems to perform ADL. Previously, the sample of OA was relatively homogeneous with high ADL scores. Therefore, we recruited OA from a fall prevention project with a varying level of physical functioning (varying scores on clinical ADL-related tests). We hypothesize that this group of OA with ADL issues would be highly influenced by the background motion (H2). Finally, we wanted to investigate the influence of dual tasks on the performance of OA with ADL issues. In our previous work (de Dieuleveult et al., 2018), the two additional tasks did not influence the illusion effect of YA and OA without ADL issues. We hypothesize that OA with ADL issues would be more influenced by the additional tasks as compared to YA (H3) and that OA with ADL issues would show differences in illusion effect between single and dual task conditions that could be associated with a decline in compensatory mechanisms that are still effective in OA without ADL problems (H4).

## Materials and Methods

### Participants

Twenty-four OA (70–88 years old, mean age 75.9 ± 4.65 years, 14 women) and nineteen YA (20–32 years old, mean age 25.6 ± 3.52 years, 12 women) participated in the study. Younger participants were recruited from the TNO participant pool (Soesterberg, Netherlands) and older participants were recruited from a fall prevention program in the health center Marne (Amstelveen, Netherlands). Participants self-reported being right-handed and having normal or corrected-to-normal vision (participants were asked to use their normal vision aids if needed). Participants’ hearing was checked by the examiner by asking them whether they could distinguish a high from a low tone used later in the experiment. Participants self-reported not to have vestibular or balance dysfunction, psychiatric symptoms, or musculoskeletal or neurological problems. They self-reported to be in relatively good health during the 2 weeks prior to the experiment and on the day of the experiment. The Mini Mental State Examination (MMSE) was used as a screening test for cognitive impairments. A cut off score of 24 or lower was used for exclusion (Dick et al., 1984).

### Task

The interception task was the same as the task described in de Dieuleveult et al. (2018). In the baseline condition, participants sat on a chair. The participants were asked to intercept, as quickly and as accurately as possible, moving targets that had disappeared after 150 ms, with the tip of their right index finger. If participants hit the target correctly, it reappeared on the screen in green and stayed still at the position of the hit. If they missed the target, it reappeared in red on the screen and moved in the opposite direction of the error. For instance, if the participant hit a position on the screen that was below the actual position of the target and too much to the left, the target moved upwards to the right. This feedback informed participants about their performance in the task and might help them learn to ignore the motion of the background. If they did not tap at all, the trial was counted as a no tap trial and the participant had to put his index finger again on the home button in order to start a new trial. The balance and counting conditions included a secondary task. In the balance condition, participants were to keep balance, standing on a block of foam rather than sitting (see below in the *stimuli and materials* section for more information on the foam). In the counting condition, participants were sitting, as in the baseline, but had to count the number of high and low tones that they heard during the experiment.

### Stimuli and Materials

In contrast to the original study (de Dieuleveult et al., 2018) the interception task was now performed on a 12,3 inch tablet rather than a 49 inch screen. Parameters were scaled down from the original experiment to best fit the tablet’s screen (see details in Table 1). Furthermore, in this experiment, the target was moving in three rather than five different directions (see below in the Stimuli and Materials section for further details).

**TABLE 1:** Differences in parameters between the original experiment (de Dieuleveult et al., 2018), and the current experiment.

**Parameters**		**Original**	**Current**
		**experiment**	**experiment**
Screen	Size (inch)	49	12,3
	Resolution (pixels)	600 × 800	2736 × 1824
Home position	Size (cm)	4	1,6
	Coordinates (cm)	(0, −30)	(0, −6)
Target	Size (cm)	6	2
	Coordinates at start (cm)	(0, 20)	(0, 8.65)
	Vertical velocity (cm/s)	50	12
	Horizontal velocities (cm/s)	−24, −12, 0, 12, 24	−7.2, 0, 7.2
Back- ground	Size squares (cm)	5	2
	Horizontal velocity (cm/s)	12	9, 6

**FIGURE 1:**
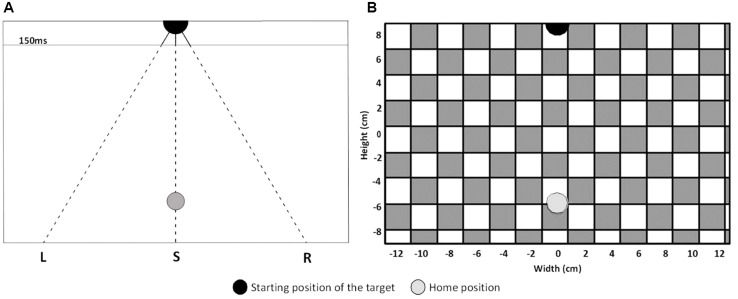
**(A)** Schematic lay-out of the stimuli for left (L), straight (S) and right (R) target’s direction of motion. The target is depicted at its appearance position as a black disc. The finger’s home position is represented by the gray disc. The solid lines represent the part of the path where the target was visible and the dashed lines represent the part of the path where the target was invisible. The horizontal line indicates where the targets disappeared after 150 ms (target has traveled 1,6 cm). **(B)** Depiction of the experimental display. As in **A**, the black and gray discs represent the starting position of the target and the home position. The gray and white squares represent the background.

During the experiment, the stimuli were presented on a 12,3-inch tablet (Microsoft Surface, size: 29,21 × 20,14 cm, resolution: 2736 × 1824 pixels) positioned on a table in a vertical orientation but tilted backward by 30°. The eyes of the participants were at a distance of approximately 60cm from the screen during the experiment (so 1cm is about one degree of visual angle). For the balance condition, where participants were standing instead of sitting, the height of the tablet was increased to keep the height of the shoulders relative to the tablet approximately the same across conditions. To start each trial, participants had to place their index finger on the home position, a green disc with a diameter of 1.6 cm situated 6 cm below the center of the screen [i.e., at coordinates (0,-6); see Figure 1 for an overview of the stimulus lay-out]. After a random time between 600 and 1200 ms, the target, a black disc with a diameter of two centimeters, appeared 8.65 cm above the center of the screen (0, 8.65) and moved toward the bottom of the screen. The target moved with a vertical velocity of 12 cm/s and one of three different horizontal velocities (−7.2, 0, or 7.2 cm/s). The three different direction of target motion are referred to as “S” for targets moving straight downwards, “L” for targets deviating to the left, and “R” for targets deviating to the right. The targets were visible for 150 ms and then disappeared. The disappearing points relative to the center of the screen (0,0) were: (−0.51, 7.05), (0.00, 7.05), and (0.51, 7.05). The targets and the home position were presented on a full screen background of white and blue squares (2 cm sides) that formed a checkerboard. The background started to move at 9.6 cm/s to the right or the left as soon as the target appeared. Auditory stimuli were presented to the participants by a computer through speakers situated behind the tablet. The computer presented sequences of high (1 kHz for 500 ms, 40% of the tones) and low tones (250 Hz for 100 ms, 60% of the tones). The intervals between the tones were randomly generated between 2 and 6 s. The tones were present during the three conditions of the interception task, but participants only had to pay attention to them in the counting condition. The block of foam used in one of the pretests and in the balance condition had a length and width of 40 cm, a height with no load of 15 cm, a height of about 10 cm when compressed by the weight of a participant, and a density of 35 kg/m^3^.

### Procedure

The session started with four standardized clinical tests used to estimate the mental and physical functioning of participants. The Mini-Mental State Examination (MMSE) (Dick et al., 1984), a 30 points questionnaire comprising short questions and simple tasks, was used to screen for cognitive impairments (cut off score of 24). The modified-Clinical Test of Sensory Interaction and Balance (m-CTSIB) (Shumway-Cook and Horak, 1986; Horn et al., 2015), a performance test in which participants have to stand on a firm and a foam surface with their eyes open or closed for 30 s each, was used to test sensory integration and balance deficits. The block of foam mentioned in the *Stimuli and Materials* section was used to perturb balance in the m-CTSIB. This test was also used to assess if the participants were fit enough to perform the balance condition safely. In order to participate in this condition, they had to be able to stand on the foam with their eyes open for 30 s without losing balance. The Short Physical Performance Battery (SPPB) (Guralnik et al., 1994; Pavasini et al., 2016) was used to assess lower limb physical functioning. It includes balance tests (side-by-side stand, semi tandem stand and tandem stand), chair stand tests and gait speed test (the ten meters gait speed test was used instead of the three- or four-meters tests). Finally, the Nottingham Extended ADL scale (NEADL) (Nouri and Lincoln, 1987) was used to assess difficulties in performing the ADL. This test is more precise than the Instrumental ADL scale (Lawton and Brody, 1969) used in the original experiment (de Dieuleveult et al., 2018). This questionnaire includes 22 questions separated in four categories, mobility (six items), kitchen (five items), domestic (five items), and leisure (six items) activities. Items can be rated from 0 (not capable of performing this activity) to 3 (easy to perform) as compared to eight items rated 0 (not capable of performing this activity) to 1 (capable of performing this activity) in the Lawton and Brody scale.

The test on the tablet was preceded by a presentation of standardized instructions by means of a PowerPoint presentation explaining the entire task and procedure to the participants. The examiner ensured that participants were able to hear the tones before starting the experiment. Participants first performed a practice session in the sitting position consisting of six trials with the target remaining visible (two trials for each of the three possible directions of target motion in randomized order) and randomized trials with the target disappearing after 150 ms until participants felt comfortable with the task. When the participants indicated that they understood the task, the experiment started. The three experimental conditions (baseline, balance, counting) were presented in random order. Each condition was presented in a block consisting of 57 trials: three practice trials without any background motion at the start (one trial per direction of target motion, presented in random order), followed by 54 experimental trials. In the 54 experimental trials, the background moved to the left in 27 trials (nine trials per direction of target motion) and the background moved to the right in the other 27 (nine trials per direction of target motion). The three blocks were separated by short breaks. Participants could take breaks at any time during the experiment. Participants were asked to report the number of high and the number of low tones they heard during the counting block at the end of it so that the examiner had an impression as to whether they adhered to the secondary task. Participants were considering adhering if they gave a plausible answer even if it was not the correct one. The data of the participants, who reported that they did not count or gave an answer very far from the correct one, were excluded from the data analyses for the counting condition.

### Data Analyses

#### Replication of the Original Experiment on a Tablet With YA

The analysis of the replication of the original experiment on a tablet will be done only in the YA group because they were recruited from the same population enabling the comparison between the two studies. The participating YA were recruited through the same participant pool as in the original experiment.

#### Direction Error and Illusion Effect

**FIGURE 2:**
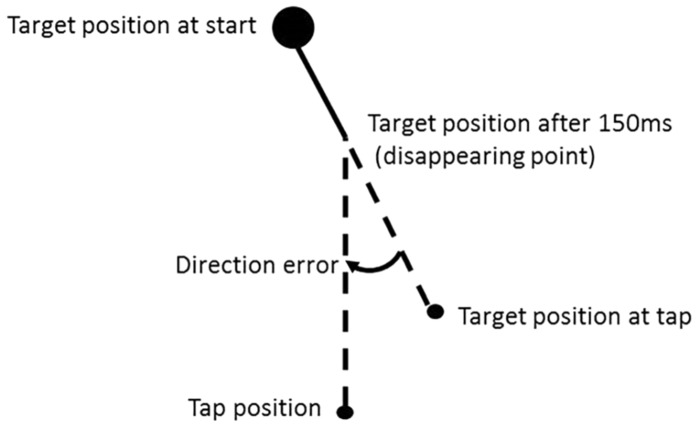
Definition of the direction error according to the tap position. The direction error is the angle in degrees between the line from the target position at start to the target position at the time of tap and a line from the position of the target when it disappeared to the tap position. The direction error were assigned a negative value when, as in the example here, the hit position was to the left of the target position at the time of tap (de Dieuleveult et al., 2018).

The direction error is defined as the angle in degrees between the direction that the target was moving in and the direction that the participant considered it to be moving after disappearing according to the position of tap (see Figure 2). When the tapping position was to the left compared to the actual position of the target at tap (as shown in the example given in Figure 2), the direction errors were assigned a negative value; if it was on the right, the direction error were assigned a positive value. The mean direction error was calculated for each participant, experimental condition and direction of background motion. The direction error is our main dependent variable. The illusion effect is defined as the direction error for left background motion effect minus the direction error for the right background.

#### Hit, Miss, and “No Tap” Trials

Trials were considered to be hit trials if the participant’s finger hit the screen within 2cm from the center of the target. If the participant’s hit exceeded the 2 cm range, the trial was considered a miss trial. Trials in which participants did not hit the screen at all were considered “no tap” trials.

#### Average Time to Tap

The time to tap is the time between the appearance of the target and the moment that the participant’s finger hit the screen.

#### Statistical Analyses

The MATLAB functions *qqplot* and *vartest2* were used to assess the normality of distribution of the residuals and the equality of the variances between the groups and conditions. With normal distributions and variances, one-way ANOVAs and paired t-tests were used to evaluate interaction effects of the different independent variables. With non-normal distributions and/or variances, non-parametric tests were used: the Mann–Whitney *U*-test for differences due to age (unpaired samples) and the Wilcoxon signed-rank test for differences due to conditions (paired samples). Effects are considered to be significant if *p* < 0.05. The chi-square test was used to test whether groups of participants differed with respect to gender.

The analysis of the replication of the original experiment on a tablet (H1) will be done only in the YA group. These participants were recruited from the same population and through the same participant pool as in the original experiment enabling the comparison between the two studies. To investigate task performance of OA with problems to perform ADL (H2), the performance of this population in the task will be compared to the performance of (relatively fit) OA of the original experiment. To investigate the influence of dual tasks on the performance of OA with ADL issues, the performance of this population in the task will be compared to YA (H3). We also looked at the different responses that OA with ADL issues and (relatively fit) OA may have in the task (H4). To answer these four hypotheses, we tested for effects of direction of background motion (left and right), experimental condition (baseline, counting and balance) on the interception task variables between the groups. We compared whether the effects in the balance condition were different than in the baseline condition and whether the effects in the counting condition were different than in the baseline condition.

## Results

YA (*n* = 19) performed all the pretests and conditions of the experiment. The majority of the OA (*n* = 15) were able to perform all conditions of the experiment (baseline, balance and counting). Five participants were unable to stand on the foam with eyes open for 30 s in the m-CTISB and therefore were not asked to perform the balance condition. Three of these five participants also failed at counting the tones in the counting condition. Four additional OA did not count the tones in the counting condition but did perform the m-CTSIB properly. The data of the seven participants that did not properly perform the counting task (all of them OA) were not included for the analysis of the counting condition results. Additionally, two OA were excluded from the analyses because their performance on the interception task was very different from the other participants (high number of no tap trials or long times to tap). One of these two participants performed only the baseline condition, the other one performed the baseline and counting condition. In total, 19 YA were included for the analyses of the data for the three experimental condition, 22 OA in the baseline condition, 19 OA in the balance condition and 16 OA in the counting condition.

### Replication of the Original Experiment on a Tablet in YA (Hypothesis 1)

**FIGURE 3:**
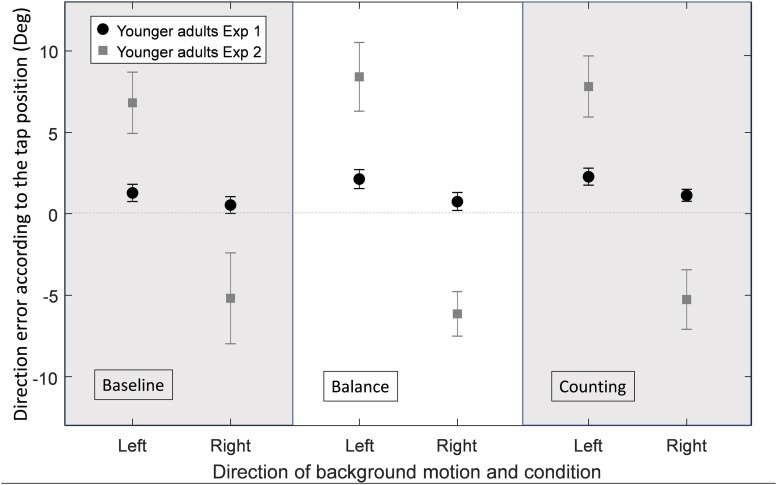
Average direction error in degrees for YA in the original experiment (Exp1, *n* = 19) and in the current experiment (Exp2, *n* = 19), for each experimental condition (baseline, balance and counting) and for each background direction of motion (left or right) merged between the different directions of the target’s motion (horizontal velocities: −7.2, 0, and 7.2 cm/s). Error bars represent the standard error of the mean between subjects.

The group of YA in the current experiment was not different from the group of YA of the original one in terms of age [*F*(1, 36) = 0.39, *p* = 0.537], gender [χ^2^(1) = 0.43, *p* = 0.511], m-CTSIB scores (*U* = 180.5, *p* = 0.344), gait speed [*F*(1, 36) = 0.08, *p* = 0.785], chair stand speed [*F*(1, 36) = 1.87, *p* = 0.180], and MMSE scores [*F*(1, 36) = 2.23, *p* = 0.144]. These results therefore allowed comparison of the current experiment with the original one for YA. Table 2 shows the comparison between the lab setup of the original experiment to the mobile setup of the current experiment for the different performance measures, for YA. Figure 3 shows an effect of the background motion in YA with direction errors more positive for a left background motion and more negative for a right background motion in all three conditions (baseline, balance and counting). This effect of the background motion is larger than the one we found in the original experiment (Student’s paired *T*-Tests: all *p* < 0.01). These results were not different between the three conditions for YA (Student’s paired *T*-Tests: all *p* < 0.01). The main performance patterns (i.e., the presence and direction of the illusion effect and the direction error) are similar in both experiments, but the tendency to hit toward the center, the lower percentage of hits and longer times to tap in baseline and balance condition (Table 2) suggest that task difficulty increased in the tablet set-up compared to the original laboratory setup.

**TABLE 2:** Comparison of different results of the current experiment (*n* = 19) with the results of the original experiment (*n* = 19) in YA (illusion effect, target direction effect, percentage of hits, number of no tap trials and time to tap).

**Measure**	**Effect**	***p*-value of Mann–Whitney *U*-tests (one for each of the three conditions)**
Illusion effect	Replicated. The direction of the Illusion effect is the same.	All *p* < 0.001
	However its size is significantly larger as compared to the original experiment.	
Target direction effect	Replicated. The target direction effect is similar to that of the original experiment: participants tapped following the target direction of motion.	All *p* < 0.001
	However, the current experiment showed a (non-significant) tendency of participants to tap toward the center of the screen that was not found in the original experiment.	
Percentage of hits	Not replicated. The average percentage of hits in the current experiment was significantly lower as compared to the original experiment	All *p* < 0.01
Number of no tap trials	Replicated. The numbers of no tap trials were not significantly different between the current and the original experiment	All *p* > 0.05
Time to tap	Partly replicated. Times to tap in the baseline and balance conditions were longer as compared to the original experiment but not different between the two experiments for the counting condition	Baseline and balance conditions, *p* < 0.01; Counting condition, *U* = 180.5, *p* = 0.102

### Performance of OA With ADL Problems in the Baseline Condition as Compared to (Relatively Fit) OA Measured in the Original Experiment (Hypothesis 2)

As expected and intended, the group of OA was different from the group used in the original experiment, with OA in the current experiment having a higher mean age [*F*(1, 42) = 25.55, *p* < 0.001] and lower pretests scores: m-CTSIB scores (*U* = 242, *p* < 0.001), gait speed scores (*U* = 231, *p* < 0.001), chair stand speed (*U* = 264, *p* = 0.004), and MMSE scores [*F*(1, 42) = 11.12, *p* = 0.002]. The two groups of OA were not different in terms of gender [χ^2^(1) = 2.32, *p* = 0.128].

#### Illusion Effect

**FIGURE 4:**
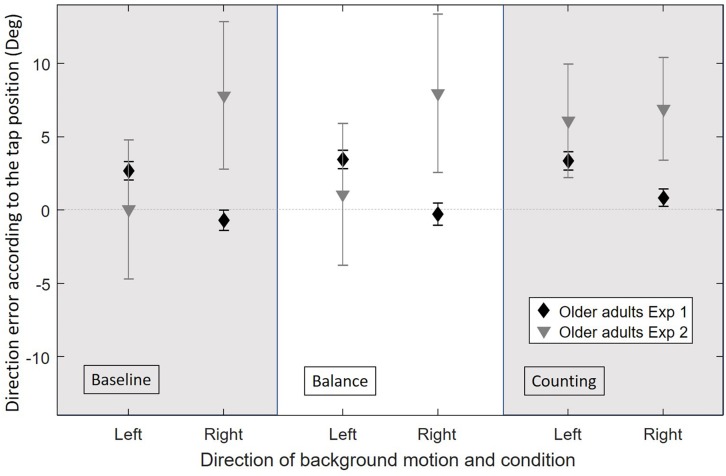
Average direction error in degrees for OA in the original experiment (Exp1, *n* = 19) and in the current experiment [Exp2, (baseline: *n* = 22, balance: *n* = 19, counting: *n* = 16)], for each experimental condition (baseline, balance, and counting) and for each background direction of motion (left or right) merged between the different directions of the target’s motion (horizontal velocities: −7.2, 0, and 7.2 cm/s). Error bars represent the standard error of the mean between subjects.

Figure 4 shows the direction errors of the OA’ taps according to the experimental condition and background direction of motion for the original and current experiments. For H2 we focus on the results of OA. The figure suggests a reverse effect of the background for the OA in the baseline condition, however, the background motion did not affect the direction errors significantly (*Z* = −0.146, *p* = 0.884). These results are different than the ones found for (relatively fit) OA in the original experiment in which OA had a large illusion effect caused by the background motion, larger than the effect observed for YA.

The absence of an illusion effect in OA found in the current experiment was not in accordance with our hypothesis (H2). The variability in the direction errors of OA is relatively large, which matches the impression of the leader of the experiment, who observed that some participants seemed to tap following the background motion rather than the target’s direction of motion, and that other participants seemed to tap continuously toward the center of the screen instead of following the target’s direction of motion. These patterns may cancel each other and result in the lack of an overall effect. Therefore, we decided to look at the illusion effect for individual participants. The results are presented in Figure 4.

**FIGURE 5:**
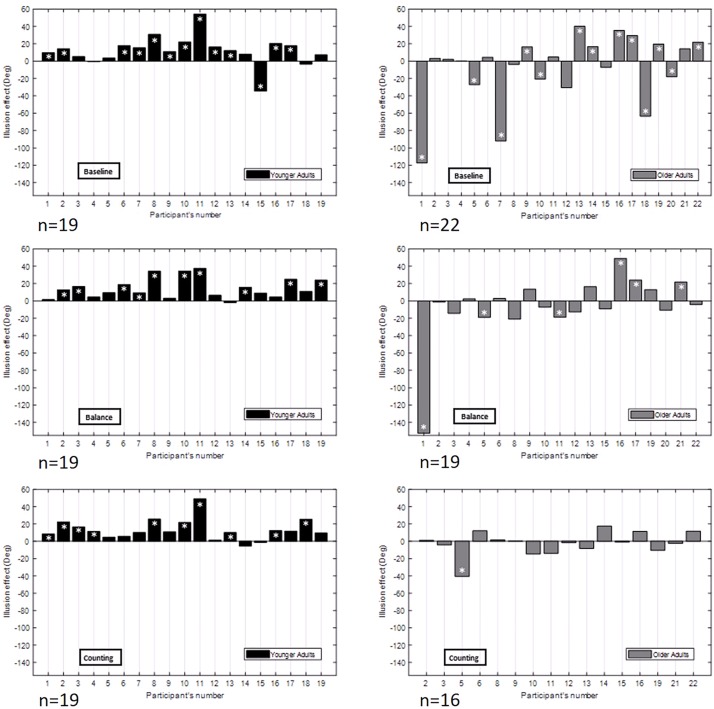
Illusion effect (left minus right background motion effect) in degrees for each participant in each age group [YA (*n* = 19), OA (baseline: *n* = 22, balance: *n* = 19, counting: *n* = 16)] and each experimental condition (baseline, balance, and counting). Younger adults are represented in black. Older adults are represented in gray. The white asterisks represent significant differences between the left and right background motion effect.

Figure 5 shows the illusion effect for each participant in each experimental condition. In the baseline condition, YA tended to have a positive illusion effect, meaning that they tapped more to the left for a right background motion and more to the left for a right background motion. This is in accordance with results found in the original experiment and in the literature (de Dieuleveult et al., 2018). The group of OA showed larger variability. In the baseline condition, three different patterns of OA could be observed with at least 6 participants in each: participants with an illusion effect similar but larger to the one found in YA (“over integration” pattern), participants with no illusion effect (“minimal use of visual information” pattern), and participants with a reverse illusion effect who tapped more to the right for a right background motion and more to the left for a left background motion (“dragged by the background” pattern).

These results are different from the results of the original experiment for OA. In the baseline condition of the original experiment, only one OA showed a reverse effect of the illusion (“dragged by the background” pattern). All the other OA showed either a positive illusion effect (“over integration” pattern) or no effect of the illusion (“minimal use of visual information” pattern).

#### Performance of OA With the Three Patterns in the ADL-Related Pretests and in the Interception Task in the Baseline Condition

The results above showed three different patterns of tapping in the OA of the current experiment (“over integration” pattern, “minimal use of visual information” pattern, and “dragged by the background” pattern). The ADL-related pretests results were not significantly different between these three patterns of OA in the baseline condition (Mann–Whitney *U*-test: all *p* > 0.05). However, the “over integration” pattern of OA had less no tap trials than the “dragged by the background” pattern (*W* = 42, *p* = 0.029) and had longer times to tap as compared to the “minimal use of visual information” pattern (*W* = 80, *p* = 0.031) in the baseline condition. The other results of the interception task were not significantly different between the three patterns of OA in the baseline condition.

#### Percentage of Hits, Number of No Tap Trials and Time to Tap

**FIGURE 6:**
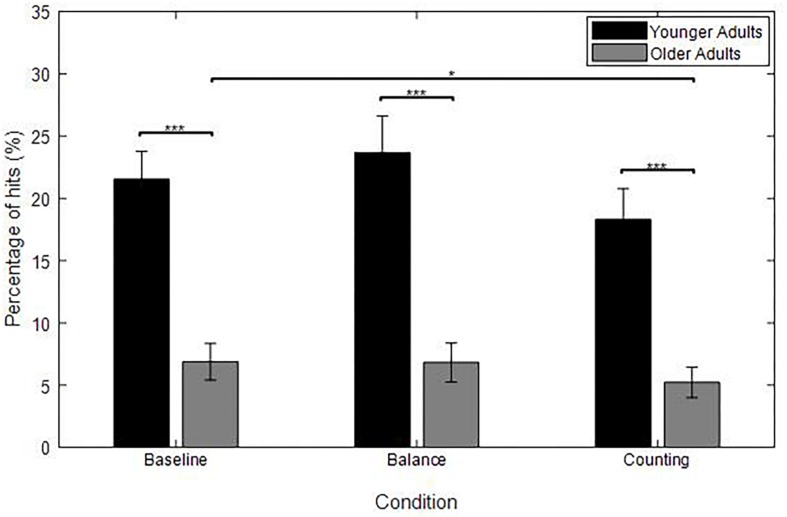
Average percentage of hits for each group [YA (*n* = 19), OA (baseline: *n* = 22, balance: *n* = 19, counting: *n* = 16)] and for each experimental condition (baseline, balance, and counting); averaged over the target’s motion direction (horizontal velocities: −7.2, 0, and 7.2 cm/s). Error bars represent the standard error of the mean between subjects. Significant differences are represented with asterisks in the figures; ^*^p < 0.05; ^∗∗∗^*p* < 0.001.

Figure 6 shows a higher percentage of hits for YA compared to OA in the baseline condition (*U* = 209, *p* < 0.001). This difference between YA and OA followed the same trend as the one found in the original experiment.

OA had a higher number of no tap trials compared to YA in the baseline (*U* = 209, *p* < 0.001). This difference between YA and OA followed the same trend as the one found in the original experiment.

The time participants took to hit the screen was different between the age groups in the baseline condition with OA being slower than YA (*U* = 209, *p* = 0.043). This trend was different than the results of the original experiment that showed no difference between YA and OA in terms of times to tap (de Dieuleveult et al., 2018).

### Influence of Dual Tasks on Interception Task Performance for YA and OA With ADL Problems (Hypothesis 3)

#### Illusion Effect

As seen in Figure 4, the overall illusion effect was not different between the three conditions for OA (Wilcoxon signed-rank test on the illusion effect: all *p* > 0.05). The illusion effect was significantly smaller for OA as compared to YA in the balance and counting conditions (respectively, *U* = 180.5, *p* = 0.007 and *U* = 152, *p* = 0.006). This did not reach significance in the baseline condition (*U* = 209, *p* = 0.078).

The introduction of additional tasks had an effect on the number of OA showing the three illusion effect patterns (“over integration” pattern, “dragged by the background” pattern, and “minimal use of visual information” pattern). For this analysis, only the data of OA that performed the three experimental conditions were taken into account to compare the changes of patterns in the participants. In the balance condition, the percentage of participants with the “minimal use of visual information” pattern was larger than the one for the baseline condition (73.3% as opposed to 53.3%) decreasing the percentage of participants with the “over integration” pattern as compared to the baseline condition (13.3% as opposed to 33.3%). The percentage of participants with the “dragged by the background” pattern was the same between the baseline and balance conditions (13.3%). In the counting condition, all of the OA showed the “minimal use of visual information” pattern except one participant that had a reverse illusion effect. This condition decreased the percentages of participants with the “dragged by the background” pattern (6.7%) and with the “over integration” pattern (0%) and increased the percentage of participants with the “minimal use of visual information” pattern (93.3%) as compared to the baseline and balance conditions.

#### Percentage of Hits

Figure 6 shows a higher percentage of hits for YA compared to OA in each condition (Mann–Whitney *U*-test: all *p* < 0.001). This graph also shows that OA hit less targets in the counting condition compared to the baseline (*W* = 89, *p* = 0.020).

#### Number of No Tap Trials

OA had a higher number of no tap trials compared to YA in all three conditions (Mann–Whitney *U*-test: all *p* < 0.001). For both groups the number of no tap trials was higher for the counting condition compared to the baseline condition (YA: *W* = 0, *p* = 0.03; OA: *W* = 7.5, *p* = 0.005).

#### Time to Tap

The time participants took to hit the screen was different between the age groups in the baseline and balance condition with OA being slower than YA (baseline: *U* = 209, *p* = 0.043; balance: *U* = 180.5, *p* = 0.018). This was not observed in the counting condition (*U* = 152, *p* = 0.253). The times participants took to hit the screen were not different between the baseline condition and the two other experimental conditions in either of the age groups (Wilcoxon signed-rank test: *p* > 0.05).

### Influence of Dual Tasks on Interception Task Performance for (Relatively Fit) OA Measured in the Original Experiment and OA With ADL Problems (Hypothesis 4)

#### Illusion Effect

We observed that the way the types of illusion effects are distributed across participants depends on the condition in OA in the current experiment (right of Figure 5), while it was virtually constant across conditions for the (relatively fit) OA in the original experiment (Baseline: 47.4% “over integration” pattern, 47.4% “minimal use of visual information” pattern, 5.2% (one participant) “dragged by the background” pattern; Balance: 47.4% “over integration” pattern, 52.6% “minimal use of visual information” pattern). The increased cognitive demand of the counting task had the same effect in the two experiments increasing slightly the number of participants with the “minimal use of visual information” pattern and decreasing slightly the number of participants with the “over integration” pattern compared to the baseline (Counting: 31.6% “over integration” pattern, 68.4% “minimal use of visual information” pattern, 0% “dragged by the background” pattern).

#### Percentage of Hits, Number of No Tap Trials and Time to Tap

OA in both experiments hit less targets in the counting condition as compared to the baseline condition.

OA in both experiments had more no tap trials in the counting condition compared to the baseline condition.

The times to tap for OA in the current experiment were not different between the conditions. These results are different from what we found in the original experiment where OA hit the screen faster in the balance condition and slower in the counting condition compared to the baseline condition.

## Discussion

This study first aimed at investigating whether we can replicate the results found in an earlier experiment in which bulky lab-grade equipment was used with a mobile setup in order to develop a diagnostic tool of sensory integration issues for OA. Second, this study aimed at investigating the differences between healthy YA and OA with varying levels of physical functioning in the interception task.

### Replication of the Original Experiment on a Tablet in YA (Hypothesis 1)

Our first hypothesis was that the effect of the moving background would be similar in both experiments (H1). As Table 2 indicates, the main effects were replicated, but there were also differences between both experiments: taps deviated to the center of the screen, the percentage of hits was lower, and the times to tap the screen were longer in two of the three conditions as compared to the original experiment. Differences in task difficulty may have caused these dissimilarities. By scaling down the setup to the tablet size, three adjustments were necessary that likely increased the general task difficulty: target size, trajectory visibility, and target speed as discussed below. We argue that the increased task difficulty is the underlying cause of all above mentioned effects.

The size of the target was significantly smaller in the current experiment compared to the original one with a diameter of two centimeters (2° of visual angle) instead of six centimeters (6° of visual angle) while the distance between the eyes and the screen was about the same (see Table 1). This may have increased the difficulty of the overall task for all participants. Indeed, it is known that a decrease in target size negatively impacts its detection (Kincaid et al., 1960; Johnson et al., 1978). Additionally, the size of the target was larger in proportion to its visible traveled trajectory. In the original experiment, the five different direction of target motion were easily distinguishable. In the current experiment, the deviations were smaller and, with the addition of the target being proportionally larger, it might have been harder to distinguish the three directions of target motion because of larger overlapping in trajectories at the beginning of the path.

Another difference that may have been essential was the changed speed of the target; 12 cm/s in the current as opposed to 50 cm/s in the original experiment (de Dieuleveult et al., 2018). The larger illusion effect with slower targets is in accordance with results found by Brenner and Smeets (2015) who showed that the influence of the moving background was larger for slower targets (Brenner and Smeets, 2015). The exact cause of this effect has to be examined in order to understand why the influence of the moving background is smaller when the target or, associated to that, the hand is moving faster. Apart from differences in task difficulty, we cannot exclude that other, unknown differences between the two experiments have played a role, such as environmental and demographical differences (besides gender and age which we checked for).

### Performance of OA With ADL Problems in the Baseline Condition as Compared to (Relatively Fit) OA Measured in the Original Experiment (Hypothesis 2)

We hypothesized that the OA in the current experiment (with ADL problems) would be more influenced by the background motion. In contrast to what we hypothesized, the study shows that the OA in the current study showed three different patterns of illusion effect: a (large) illusion effect (“over integration” pattern), a reverse illusion effect (“dragged by the background” pattern) and no illusion effect (“minimal use of visual information” pattern). The “dragged by the background” pattern was not present in the (relatively fit) OA of the original experiment (only one participant in the baseline condition).

These effects may be caused by the fact that OA in the current study were less fit and therefore may have had more difficulties to properly perform the task, i.e., the effects are caused by differences in the samples of OA. In addition, task difficulty may have modulated the effects of age for specific samples. Therefore, both factors (increased task difficulty and OA group differences) are elaborated upon below.

#### Increased Task Difficulty for OA With ADL Issues Compared to (Relatively Fit) OA

Generally, performance of both YA and OA suggest that the task is more difficult in the current setup [see section Replication of the Original Experiment on a Tablet in YA (Hypothesis 1) that explain the increased task difficulty]. With advancing age, sensorimotor performance is altered. OA have, for example, difficulties in coordination, an increased variability of movement, and slower movements (Contreras-Vidal et al., 1998; Seidler et al., 2010; Lee et al., 2013). As a consequence, with age, OA are less and less able to perform accurate movements. The smaller target of the current experiment (two centimeters instead of six centimeters) required participants to be more accurate when intercepting the target as compared to the original one. These changes might have increased the difficulty of the task for OA with ADL issues compared to OA without ADL issues.

As described by Hubel and Wiesel (1962), perception of objects and motion is driven by feed forward connections between the lateral geniculate nucleus (LGN), layer IV simple cells and layer II/III complex cells leading to orientation and direction-selective receptive fields in the primary visual cortex neurons (V1) (Hubel and Wiesel, 1962; Alitto and Dan, 2010). Gamma-aminobutyric acid (GABA) mediated inhibition is necessary for neuronal responsiveness and selectivity in V1 in order to suppress neuronal responses for irrelevant stimuli (Alitto and Dan, 2010). These excitation/inhibition processes have been shown to be less effective in OA (Leventhal et al., 2003; Francois et al., 2011; McDougall et al., 2018). This may underlie our original finding (de Dieuleveult et al., 2018) that OA are less able to ignore the (irrelevant) background motion in the interception task. McDougall et al. recently discovered that the receptive fields of V1 neurons in OA could expand due to changes in GABAergic functioning leading to a summation enhancement of the neurons responses (McDougall et al., 2018). As a consequence, signals from different small moving stimuli (the target and the squares of the background in the interception task) are pooled together, leading to erroneous percept and additional motion noise (McDougall et al., 2018). The target and the squares of the background being smaller in the current experiment as compared to the original one, might have increased this summation enhancement issue and increased the difficulty of OA to dissociate these two stimuli.

Age-related decline in motion sensitivity is also known to be particularly strong for central vision as compared to peripheral vision (Wojciechowski et al., 1995; Owsley, 2011). The target in the current experiment was smaller than the one in the original experiment (2° of visual angle instead of 6°). OA had to rely more on their (compromised) central vision to distinguish the target from the background in the present study, thus increasing their tendency to tap in the center of the screen as a more probabilistic strategy, reducing the illusion effect and decreasing their percentage of hits.

#### OA Group Differences

**FIGURE 7:**
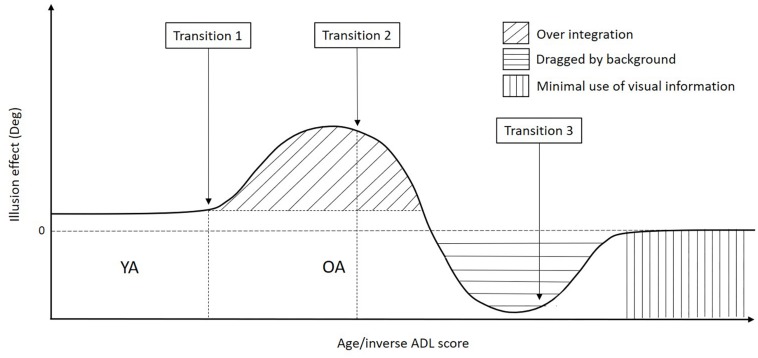
Graph representing the three hypothesized age-related transitions in patterns happening in the interception task. In the early phase of age-related changes (transition 1), there is a tendency to integrate all available information with a lack of proper weighting less reliable information (“over integration” pattern). In a second phase of the age-related process, OA become unable to downregulate the task-irrelevant background and are dragged by it, showing a reverse effect of the illusion (transition 2) (“dragged by the background” pattern). In a later phase of age-related changes, multisensory integration becomes too difficult, and older adults change their pattern again (transition 3), basically turning the interception task into Reaction Time task (“ minimal use of visual information” pattern).

Differences between the two groups of OA might add up to the increased task difficulty as a cause of differences in the task performance. Compared to the original experiment, the OA in this experiment were selected such as to also include individuals who would score below ceiling level on the conventional ADL-related tests. They were also on average older than the participants of the original experiment (mean age: 75.9 ± 4.65 years compared to 67 ± 6.40 years). Thus, the degradations described earlier (motion discrimination problems), as well as other age related decline such as increased variability of movements, slowing movements, coordination problems and balance problems (Seidler et al., 2010; Lee et al., 2013), may have become more noticeable and lead to different results between the current compared to the original experiment. The combined results of the original experiment and the current one, lead us to propose a transitional model of the aging process happening in our task (model depicted in Figure 7).

In this model, YA are depicted first. This group of participants puts a high weight on the target and small or no weight on the background, resulting in a small illusion effect caused by the moving background. In other words: YA are able to largely (but not completely) ignore the task irrelevant background motion.

The original experiment showed an increased illusion effect for OA as compared to YA. The group of OA that participated in that experiment did not have any problem to perform the ADL. Similar results were found for some of the OA in the current experiment. In our previous article, we hypothesized that the strong illusion effect was caused by OA having more trouble to ignore irrelevant sensory information in general (Teasdale et al., 1991; Berard et al., 2012; Eikema et al., 2014; McGovern et al., 2014; de Dieuleveult et al., 2017). With age, proprioceptive and vestibular information become less reliable and may force OA to rely more on vision, they put more weight on the visual cues, leading to an over integration of the background motion and larger direction error (Brenner and van den Berg, 1994; de Dieuleveult et al., 2018). Therefore, this early stage of the aging-related degradation process might be the cause of a first transition in performance patterns (transition 1 in Figure 7) with participants over-integrating the background motion as compared to YA. This is in line with previously found results of the literature that showed that OA tend to integrate all the information present in the environment (Teasdale et al., 1991; Berard et al., 2012; Eikema et al., 2014; McGovern et al., 2014; de Dieuleveult et al., 2017).

The OA in the current experiment had on average more problems to perform the ADL as compared to OA of the original experiment. They had lower scores in the pretests as well, were older, hit less targets and were slower. Some of the OA in the current experiment had similar results as the ones that participated in the original experiment (“over integration” pattern). However, some had a reverse illusion effect (tapped more to the left for a left background motion and more to the right for a right background motion, “dragged by the background” pattern) and some did not show any effect of the illusion (“minimal use of visual information” pattern).

In the second pattern described in this experiment (“dragged by the background” pattern), the OA showed a reverse illusion effect as compared to the “over integration” pattern. This effect was not observed in the original experiment. This could reveal a second pattern of the aging process happening in our task, the age-related degradations in the sensory systems might become larger and hinder participants from downregulating the task-irrelevant, but relatively salient, background. Participants put more and more weight on the background to the extent that the background outweighs the target. They start then to be dragged by the background motion that they cannot ignore and tap following this background instead of following the target’s direction of motion. These results are supported by the fact that the OA with the “dragged by the background” pattern had more no tap trials than the ones with the “over integration” pattern, showing that the task was more difficult for them.

For the last pattern (“minimal use of visual information” pattern), the illusion effect found in the original experiment was not present since these participants tapped toward the center of the screen instead of following the target or background direction of motion. These results suggest that when the task becomes too difficult, a third transition in patterns and therewith performance occur in OA (transition 3 in Figure 7). Participants don’t try to intercept the targets anymore. The interception task, which required sensory integration, became a simple RT task with participants taping in the center of the screen in a more probabilistic strategy. These results are supported by the fact that the participants with the “minimal use of visual information” pattern had shorter times to tap as compared to the “over integration” pattern, showing that they tend to tap as fast as possible. This is in line with findings of the literature that showed that OA present a slowing of the integrative systems and difficulties to integrate sensory information into the central nervous system (Stelmach et al., 1989; Teasdale et al., 1991; Furman et al., 2003; Diederich et al., 2008; Hugenschmidt et al., 2009; Stephen et al., 2010; Elliott et al., 2011; Mahoney et al., 2011; Dobreva et al., 2012; Mahoney et al., 2012; Mozolic et al., 2012; Wu et al., 2012; DeLoss et al., 2013; Guerreiro et al., 2014, 2015). This transition to a different (easier) pattern, driven by task difficulty, might be a good indicator for ADL performance.

Additionally, the OA of the current experiment were older than the ones of the original experiment, and thus might have been more affected by age-related movement deficits. Indeed, with age, OA experience a decline in sensorimotor control and functioning which can be attributed to changes in the central and peripheral nervous systems and the neuromuscular system (Seidler et al., 2010). These changes lead to motor performance deficits often associated with a diminished ADL performance (Seidler et al., 2010). These deficits might be an additional cause of difficulties to reach the screen accurately for these participants as compared to the participants of the original experiment and might add to the causes of this change in pattern.

We did not find any differences in the performance of the ADL-related pretests for the three patterns of illusion. This is not in accordance with the expected decrease in performance across the patterns. These results might be explained by the small number of participants with each of the three patterns. Additionally, as opposed to the interception task, the ADL-related pretests do not focus on vision and upper limb movements, which can explain why we found no relation between the results of the tasks.

### Influence of Dual Tasks on Interception Task Performance for YA and OA With ADL Problems (Hypothesis 3)

We hypothesized that OA with ADL issues would be more influenced by the additional tasks as compared to YA (H3). The results of the experiment did not confirm this hypothesis. Indeed, we presented two dual tasks in this experiment, one that perturbed mainly the proprioceptive and vestibular inputs (balance condition) and one that perturbed mostly cognition (counting condition). The results of Figures 4, 5 showed that these additional tasks have a negative impact on the performance of both YA and OA but did not show a larger effect of the dual tasks in OA compared to YA. This is in accordance with previously reported results showing that dual tasks decrease task performance (Redfern et al., 2001, 2009; Mahboobin et al., 2007; Bisson et al., 2014; de Dieuleveult et al., 2017).

Standing on the foam in the balance condition influenced the illusion effect in YA and OA. Indeed, this additional task increased the number of participants that tapped toward the center of the screen without following the actual direction of the target motion in both age groups as compared to the baseline condition. However, the percentages of hits, numbers of no tap trials and times to tap were not different between the balance and baseline conditions for both age groups.

The counting condition seemed to be more challenging for both age groups. In this condition, none of the OA had an effect of the background motion except one participant that had a reverse illusion effect. None of these participants had an “over integration” illusion effect. Additionally, both age groups had a larger amount of no tap trials in the counting condition as compared to the baseline condition and the OA intercepted less targets in the counting condition than in the baseline condition.

### Influence of Dual Tasks on Interception Task Performance for (Relatively Fit) OA Measured in the Original Experiment and OA With ADL Problems (Hypothesis 4)

We hypothesized that OA with ADL issues would show differences in illusion effect between dual and single task conditions as opposed to OA without ADL problems (H4). These differences could be associated with a decline in compensatory mechanisms in OA with ADL issues that are still effective in OA without ADL problems. The results of the experiment confirmed this hypothesis. Indeed, OA with ADL problems measured in the current experiment showed differences between the experimental conditions. Both additional tasks increased the number of OA with the “minimal use of visual information” pattern while only the counting condition did in the original experiment. Additionally, the group of OA in the current experiment comprised a large proportion of OA with the “dragged by the background” pattern in both the baseline and balance conditions while only one participant showed this pattern in the baseline in the original experiment.

The changes observed in OA with ADL problems in the presence of secondary tasks might reveal proprioceptive/vestibular and cognitive compensatory mechanisms that normally help to reduce deficits caused by being unable to ignore the background motion in our task. With age, these compensatory mechanisms might become no longer sufficient. This could explain the degradation in performance in the interception task observed in OA. Aging participants rely more and more on the visual system and are less and less able to ignore the task-irrelevant background and tend to be dragged by it when tapping (reverse effect of the illusion). This decline in compensatory mechanisms might then be responsible for transition 2 and could be a factor of poorer performance in the ADLs.

## Limitations

The experiment described here is a first step toward the development of a diagnostic tool. However, and especially because different response patterns in different participants were found, further experiments, with a substantially larger number of participants are necessary to validate the tool and to link participants’ characteristics (i.e., age and ADL score) to their pattern on the interception task, and to explore the modulating effect of task difficulty. For example, varying task difficulty may help to reveal individual transition points (transitions 1, 2 and 3 in Figure 7) that in turn might correlate with ADL score. Such an approach would benefit from a wide range in age and ADL issues. This way, it would be possible to identify more clearly the different key transitional points in terms of age and ADL scores, understand better the causes of these changes and choose best interventions in order to train ADL performance.

The fact that some OA tend to tap toward the center of the screen instead of following the actual direction of the target motion could also be described as non-adherence to the task. This could reflect a genuine inability to perform the task or a lack of motivation. A lack of motivation can be caused by OA who do not trust their ability to perform the task, or by annoyance by the task that they found too difficult. Tapping toward the center of the screen lessens the physical and cognitive efforts needed for the task and reduces the total time of each block of trials because participants tapped the screen as fast as possible. This is in accordance with shorter times to tap found for the “minimal use of visual information” pattern as compared to the two other groups of OA. We observed large discrepancies between the participants. Most of the OA seemed to be really motivated and appeared to do their best in the task, however, a small amount of the participants seemed annoyed by the task and wanted it to be done as soon as possible. This motivational problem is not specific of our task. Indeed, even in pencil and paper tests, motivation could be an issue and could depend on the day in which the test is performed for the same participant. In order to further investigate this, developing and testing an easier version of the task would be necessary.

## Conclusion

We transferred the previously tested interception task from a large screen to a portable tablet version. Although there were differences between both setups, the tablet version showed similar effects of the background illusion for comparable samples (i.e., the YA) and was able to reveal differences between age groups (three different patterns). This warrants further investigation of the tablet as potential tool in clinical practice, for instance by linking task performance (patterns) on the interception task to ADL scores on an individual basis.

If the transitional model is valid and specific patterns correlate well with ADL scores, it would have an impact for clinical practice. In order to train individuals to help them live independently for a longer time, clinicians would need to identify where their patients are in our transitional model. Pattern one of the aging process reveals problems in multisensory integration (more specifically in appropriately weighing sensory inputs). Clinicians would then want to decrease these problems using specific multisensory integration training programs such as training programs to ignore irrelevant information in a multisensory environment. Pattern two of the aging process reveals multisensory integration issues as well and weaknesses in sensory compensatory mechanisms. Clinicians would then want to decrease these problems using specific multisensory integration training programs, as in the first pattern of the aging process, and specific proprioceptive/vestibular and cognitive training programs to restore the compensatory mechanisms. Pattern three of the aging process reveals target detection issues and motor problems. Clinicians would then want to target these specific problems using simple detection tasks and upper limb physical training programs. The interception task would be a valuable tool to distinguish the three patterns in clinical practice.

## Ethics Statement

The experimental protocol was reviewed and approved by the TNO Internal Review Board on experiments with human participants (Ethical Application Ref: TNO-2017-015) and was in accordance with the Helsinki Declaration of 1975, as revised in 2013 (World Medical Association, 2013) and the ethical guidelines of the American Psychological Association. Participants received a monetary compensation for their participation and their travel expenses were reimbursed. All participants signed an informed consent form prior to the experiment.

## Author Contributions

AD performed the experiments, analyzed the data, and wrote the manuscript. SP performed the experiments and reviewed the manuscript. A-MB, PS, and JE reviewed the manuscript.

## Conflict of Interest Statement

The authors declare that the research was conducted in the absence of any commercial or financial relationships that could be construed as a potential conflict of interest.
